# Application of Omics in Donkey Meat Research: A Review

**DOI:** 10.3390/ani15070991

**Published:** 2025-03-29

**Authors:** Qifei Zhu, Yongdong Peng, Xiaotong Liu, Wenting Chen, Mingyang Geng, Jincheng Na, Muhammad Zahoor Khan, Changfa Wang

**Affiliations:** 1 Liaocheng Research Institute of Donkey High-Efficiency Breeding and Ecological Feeding, College of Agriculture and Biology, Liaocheng University, Liaocheng 252000, China; 2 Ili Kazak Autonomous Prefecture Livestock General Station, Ili 835000, China

**Keywords:** donkey meat, composition, health benefits, growth traits, potential genes, omics technologies

## Abstract

This review comprehensively examines the molecular basis of donkey meat characteristics and growth-associated genes, integrating findings from multiple omics approaches. This study examines the nutritional profile of donkey meat, which is rich in protein, essential amino acids, and unsaturated fatty acids. Through a systematic literature search across Web of Science, Google Scholar, PubMed, and Scopus databases (2000–2024), we collected and analyzed data from 400 research articles using predefined inclusion criteria focused on nutritional composition, omics approaches, and meat quality parameters in donkey populations. The study also evaluates various factors affecting meat quality, including breed differences, age, feeding management, and storage conditions. Advanced genomic and transcriptomic analyses have revealed numerous candidate genes, such as *ACTN3*, *BMP7*, *NR6A1*, *Wnt7a*, *HOXC8*, *LCORL*, *TPM2*, and *TPM3*, associated with growth traits and meat quality characteristics, providing valuable insights for genetic improvement programs. Furthermore, the review discusses various authentication methods for ensuring donkey meat quality and preventing adulteration, highlighting the integration of traditional and modern analytical approaches.

## Simple Summary

This review discusses the emerging scientific insights into the distinctive characteristics of donkey meat. It highlights the nutritional benefits of donkey meat, including its high protein content, essential amino acids, and healthy fats. The review delves into the molecular factors that contribute to the tenderness and flavor of donkey meat. Additionally, it explores how variables such as breed, age, and feeding practices influence meat quality. The paper also addresses methods for authenticating donkey meat products and identifies areas that require further research to enhance both production and quality for consumers.

## 1. Introduction

The donkey (*Equus asinus*), a significant member of the equine family domesticated approximately 5000 years ago, has played a pivotal role throughout human history [1]. Primarily utilized for transportation, donkeys continue to serve as essential working animals, particularly in regions of Asia and Africa [2,3]. With a global population exceeding 40 million and encompassing 185 recognized varieties, donkeys contribute significantly to regional economies through their diverse applications, including transportation, meat, skin, and milk production [4,5,6,7]. Donkey milk’s nutritional composition bears remarkable similarity to human milk, offering unique benefits, including immune system enhancement, hepatic and gastric protection, and dermatological benefits, including skin-brightening properties [8,9]. Donkey-hide gelatin (Ejiao) remains a highly esteemed health supplement and valuable component in traditional Chinese medicine, enjoying significant consumer popularity for its historically recognized health-promoting properties [10,11,12,13].

Agricultural mechanization and modern transportation have reduced the reliance on donkeys for labor, contributing to population declines in certain regions. However, their economic value has shifted toward meat and medicinal products [14,15,16]. In parts of Asia, donkey-derived products, particularly skin and meat, have become integral to the food and health industries [17,18]. Donkey meat exhibits distinctive nutritional characteristics, including higher levels of crude protein and essential amino acids compared to beef and sheep, elevated proportions of unsaturated fatty acids, and lower content of total fat, cholesterol, and calories [19]. These properties align with the “three high and three low” nutritional profile recognized by Chinese consumers [14]. The amino acid composition of donkey meat closely resembles human nutritional requirements, facilitating efficient digestion and absorption. A significant market concern is the adulteration of donkey meat with cheaper alternatives, such as horse, beef, or poultry meat. This practice, primarily motivated by financial gain, is particularly prevalent in markets where donkey meat commands premium prices due to its status as a delicacy or for its traditional medicinal applications.

The regulatory landscape for donkey products varies considerably worldwide [20,21]. Many countries have implemented restrictions on the sale of donkey meat and derived products [22]. For instance, the United States prohibits donkey meat transactions due to animal welfare and food safety considerations. Similar restrictions exist in several European countries [23,24].

In biological classification, ‘omics’ encompasses comprehensive molecular component analysis across various biological levels, from fundamental genetic materials to functional metabolites. Technological advances, including high-throughput sequencing and high-resolution mass spectrometry, have enabled deeper exploration of the biomolecular realm [25,26,27,28]. These omics analyses have found widespread application in animal research [29,30,31,32,33].

In cattle studies, omics technologies facilitate investigations into production efficiency enhancement, reproductive performance optimization, metabolic analysis, and microbiome research [34,35,36]. Similarly, omics approaches have been extensively employed in studying trait formation mechanisms, developmental patterns, and quality-related characteristics in pigs, sheep, goats, and chickens [37,38,39,40,41,42,43,44]. In recent years, the widespread application of omics technologies has helped in identifying and evaluating various animal-derived products, including meat and milk [45,46,47,48,49,50,51]. The applications of omics technologies in donkey meat research have been increasingly reported in recent years [51]. These technologies have shown promise in addressing authentication challenges in donkey products and preventing fraudulent practices.

Current applications of omics technologies in donkey meat research exhibit significant limitations despite their successful implementation across various animal studies. Two key challenges persist in this field: First, the integration of donkey genetic resources with omics technologies is inadequately defined, resulting in the underutilization of available genetic material; second, there is a significant gap in the comprehensive exploration of the applications, mechanisms, and effectiveness of omics technologies in optimizing donkey meat quality. These limitations hinder the progress of the donkey meat industry through omics-based approaches. This review seeks to address these gaps by providing a thorough analysis of donkey genetic resources, evaluating the applications of omics technologies in enhancing meat quality, and systematically reviewing the research advancements in donkey meat science to establish a strong foundation for future industrial development.

## 2. Literature Search Methodology

This review focuses on the application of various omics technologies, including genomics, transcriptomics, proteomics, metabolomics, and lipidomics, for the enhancement of donkey meat quality and growth performance traits. Keywords such as omics applications, donkey meat, growth traits, vertebrae, meat quality, meat composition, health benefits, and genetic resources were used to guide the literature search. To identify relevant content for this review, we accessed several databases, including Web of Science, Google Scholar, PubMed, and Scopus, covering articles published between 2000 and 2024. We excluded content from conference proceedings, book chapters, unpublished data, and articles published in non-peer-reviewed journals or non-Science Citation Index (SCI) journals.

## 3. Characteristics of Donkey Meat

### 3.1. Donkey Meat Compositions

The nutritional profile of donkey meat is characterized by its exceptional protein content, featuring a well-balanced amino acid composition that aligns with human nutritional requirements. Of particular significance is its rich complement of essential amino acids, including lysine, threonine, and isoleucine [52]. From a lipid perspective, donkey meat exhibits a notably low total fat content while maintaining a favorable proportion of unsaturated fatty acids. The predominant unsaturated fatty acids include linoleic acid and linolenic acid, which contribute significantly to cardiovascular health and overall human well-being [53].

The micronutrient composition of donkey meat encompasses both fat-soluble vitamins and essential minerals. The presence of vitamins A and E is complemented by substantial concentrations of mineral elements, specifically iron, calcium, phosphorus, and potassium. These nutritional components serve multiple physiological functions: supporting immune system functionality, maintaining dermal health, ensuring skeletal integrity, facilitating neural transmission, and regulating cellular osmotic balance [8,54].

The sensory characteristics of donkey meat are derived from the complex interaction between its bioactive components and a variety of volatile compounds [51,52,53]. The lipid content of donkey meat is 2–4%, and the fatty acids produced by lipid hydrolysis are oxidized to form volatile flavor substances [55,56]. Moreover, as an efficient VOC retention agent, lipids exhibit strong VOC binding characteristics due to the lipophilicity of most volatile organic compounds, which in turn creates the unique sensory characteristics of donkey meat [57]. A comparative analysis of the nutritional composition between donkey meat and other livestock species is presented in Table 1.

In order to better display the nutritional components of different meats, this paper analyzes and compares multiple varieties and multiple meat parts, with the results summarized in Table 1. Donkey meat contains approximately 23.56 g/100 g of protein, a content comparable to that of beef, potentially appealing to consumers prioritizing high-protein dietary regimens. Notably, its fat content is remarkably low at 1.77 g/100 g, representing a substantial reduction compared to that of pork (23.80 g/100 g) and mutton (8.85 g/100 g). This macronutrient distribution positions donkey meat as an optimal choice for individuals seeking high-protein, low-fat nutritional options. The vitamin B12 content in donkey meat (1.90 μg/100 g) is lower than that found in beef (6.53 μg/100 g). Nevertheless, it represents a viable supplementary source for individuals with restricted dietary patterns or insufficient vitamin B12 intake. This micronutrient is critical for neurological function and erythropoiesis, underscoring its nutritional importance. Donkey meat demonstrates favorable cardiovascular health parameters, with a cholesterol content of 66.70 mg/100 g and a polyunsaturated fatty acid to saturated fatty acid ratio of 0.73. These characteristics potentially confer cardiovascular benefits when compared to alternatives such as mutton, which typically presents higher cholesterol levels. These nutritional attributes collectively contribute to donkey meat’s emerging status as a nutritionally advantageous protein source that may satisfy both consumer preferences for macronutrient optimization and health-conscious dietary requirements.

### 3.2. Physical and Chemical Properties of Donkey Meat

Donkey meat has unique structural and biochemical characteristics, which contribute to its palatability. The muscle fiber diameter of donkey meat is small, which gives it a fresh texture, but the high density of muscle fibers brings some resistance when chewing, while still allowing consumers to enjoy the texture during the chewing process. The meat’s distinctive flavor profile is attributed to its high concentration of umami amino acids, particularly glutamic acid, combined with its unique lipid composition and metabolites [78,79]. Comparative analyses of different anatomical regions of Dezhou donkey meat have revealed significant variations in physical properties. The longissimus dorsi (LD) exhibits marked differences in color and texture compared to the gluteus maximus (GM) and biceps femoris (BF). Notable characteristics of the LD include elevated intramuscular fat (IMF) content, superior fatty acid profiles, and antioxidant parameters [55]. In comparative studies of air-dried jerky products derived from various species, donkey meat demonstrated several advantageous properties, including a lower percentage of saturated fatty acids, higher concentrations of polyunsaturated and ω-3 fatty acids, enhanced protein and essential amino acid content, and superior tenderness and consumer acceptability [19,80].

### 3.3. Health Benefits

Donkey meat’s nutritional profile makes it an exceptional protein source. Its molecular composition, rich in unsaturated fatty acids, essential amino acids, and minerals, underlies its health-promoting properties [54]. The unsaturated fatty acids present in donkey meat contribute to cellular membrane regulation and cardiovascular health by modulating membrane fluidity and permeability, reducing blood cholesterol and triglyceride levels, and supporting cardiovascular disease prevention [81,82]. Essential amino acids found in donkey meat serve as crucial building blocks for protein synthesis, playing vital roles in maintaining normal physiological functions [83,84]. These characteristics make donkey meat particularly suitable for general consumption, with special benefits for elderly individuals and those with cardiovascular conditions [8]. In order to protect the rights and health of consumers and maintain the authentic meat market, it is necessary to prevent meat adulteration.

## 4. Omic Applications in Donkey Meat Research

Multi-omics technologies have revolutionized meat science research across several domains. In quality assessment and prediction, these technologies enable comprehensive evaluation of meat characteristics, including color, tenderness, flavor, and nutritional components, through multi-level analysis encompassing genes, transcription, proteins, and metabolites, leading to the identification of quality-related biomarkers for precise quality prediction [85,86,87,88]. In production process optimization, these technologies find application in animal breeding and reproduction, providing guidance for selective breeding programs and enhancement of meat yield and quality [89,90,91]. They also facilitate investigation of molecular changes during processing and storage, enabling optimization of processing technologies and storage conditions. In terms of industrial sustainability, multi-omics approaches contribute to improved resource utilization efficiency, reduction of production costs and environmental impact, enhanced quality control and safety monitoring, and strengthened consumer confidence [87,92].

### 4.1. Proteomic Applications in Donkey Meat Research

Proteomic analysis has provided valuable insights into donkey meat characteristics. A comparative study of longissimus dorsi muscles across species identified 764 and 1024 differentially expressed proteins (DEPs) between cow-donkey and goat-donkey comparisons, respectively. These DEPs are primarily involved in amino acid and lipid metabolism pathways. The study highlighted the significance of the ACO2 protein in lysine synthesis, corresponding to higher lysine content in donkey meat. Additionally, differential expression was observed in proteins IDH1, GSR, and PGD (upregulated) and Lap3 (downregulated) in glutamate metabolism [93]. Further proteomic research using data-independent acquisition (DIA) methodology revealed 111 and 127 differentially abundant proteins (DAPs) in semitendinosus/longissimus muscle (ST/LT) and gluteus maximus/longissimus muscle (GM/LT) comparisons, with involvement in phospholipase D, MAPK signaling, and fat digestion pathways. Specific correlations were found between GnRH/MAPK signaling (ST/LT) and fat metabolism (GM/LT) with meat quality parameters [94]. Based on DIA analysis of donkey gluteus superficialis (WG), longissimus pectoralis (WLT) and semitendinosus (WS), 189 and 384 differentially expressed proteins (DEPs) were found between WG/WLT and WS/WLT groups, respectively, and their regulatory pathways were significantly involved in intramuscular fat deposition, protein and amino acid metabolism [95]. The integration of proteome and transcriptome data has revealed expression patterns from gene to protein levels, particularly in dehydrogenase genes’ involvement in oxidoreductase activity and various metabolic pathways, providing mechanistic insights into donkey meat’s flavor characteristics [93].

### 4.2. Lipidomic Applications in Donkey Meat Research

Lipidomics, as a powerful analytical approach, has proven invaluable in deciphering complex lipid compositions, fatty acid profiles, oxidative stability, and shelf-life characteristics. It has been effectively applied to study the lipid-related aspects of donkey meat, providing crucial insights into these areas. This methodology facilitates the identification of lipid-based biomarkers for quality assessment, elucidates the relationships between lipid components and meat flavor, and ensures product authenticity and safety. Lipids serve not only as fundamental energy and nutritional sources but also play critical roles in food quality and human health. The IMF content has been identified as a crucial determinant of meat quality. Through LC-MS-based lipidomics analysis, researchers have demonstrated that the longissimus dorsi muscle (LDM) contains higher IMF content compared to the buttock muscle (RM) and hamstring muscle (HM). This analysis has also revealed key metabolic pathways associated with IMF variations, including glycerolipid (GL), glycerophospholipid (GP), and sphingolipid (SP) metabolism [78]. Comparative lipidomics analysis between IMF and visceral adipose tissue (VAT) has revealed higher percentages of 18:1 in triglycerides (TGs), phosphatidylcholine (PC), and phosphatidylethanolamine (PE) in LDM compared to VAT [96]. This variation is attributed to differences in cell type and fat deposition rates [97], suggesting the enhanced capability of LDM in cholesterol regulation and cardiovascular health protection [98]. Comprehensive analysis of Sanfen and Wutou donkey meat identified 1101 lipid molecules across 13 subclasses, with phospholipids constituting 61.87% of the total lipid content, significantly exceeding levels found in pork and chicken. UHPLC-ESI-MS analysis has established correlations between lipid profiles and volatile organic compounds (VOCs) across different meat species. Research has demonstrated that PUFA-rich lipids serve as essential precursors for flavor compound formation [99,100]. Specific triglycerides, notably TG (16:1_18:1_18:2) and TG (16:0_16:1_18:2), have been identified as key compounds for VOC retention in boiled donkey meat, while phospholipids and their derivatives play crucial roles in the cooking process [57]. Certain lipid species, such as PC (18:3e_16:0) and MePC (31:0e), serve as effective markers for distinguishing between raw (RDM) and cooked donkey meat (CDM) [57,100].

### 4.3. Metabolomic Applications in Donkey Meat Research

Flavor perception, encompassing both taste and aroma, significantly influences consumer evaluation and purchasing decisions [101]. Electronic nose analysis has revealed distinct flavor profiles between donkey meat and other species, with aldehydes derived from lipid autooxidation playing a crucial role in flavor differentiation. Studies have identified oleic and linoleic acids as key unsaturated fatty acids influencing donkey meat flavor, with notably higher oleic acid content in donkey neck meat compared to beef and sheep [102,103]. GC-IMS spectroscopy and fingerprint analysis have demonstrated significant variations in ketones, alcohols, and aldehydes both within and between donkey breeds [104]. Free amino acids contribute to flavor development through Maillard reactions and Strecker degradation. Donkey meat exhibits higher total amino acid content compared to other species, with predominant amino acids including alanine, lysine, glutamic acid, glycine, and serine. The elevated expression of key genes such as ALDH9A1, PGD, FAHD1, and AOC1 in amino acid metabolic pathways may contribute to the distinctive taste profile of donkey meat [93,105]. Metabolomic analysis has identified 37 differential metabolites between cooked (CDM) and raw donkey meat (RDM), with maltotriose, L-glutamic acid, and L-proline contributing to unique umami and sweet flavors [101]. Nine metabolites, including L-glutamic acid, γ-aminobutyric acid, and butane-1,2,3,4-tetraol, serve as potential biomarkers for distinguishing between raw and cooked meat [96]. Analysis of volatile compounds using SPME and GC-MS has revealed the prevalence of hydrocarbons, alkanes, and alcohols, while aging processes influence key quality parameters including tenderness and polyunsaturated fatty acid content [99,106].

**Figure 1:**
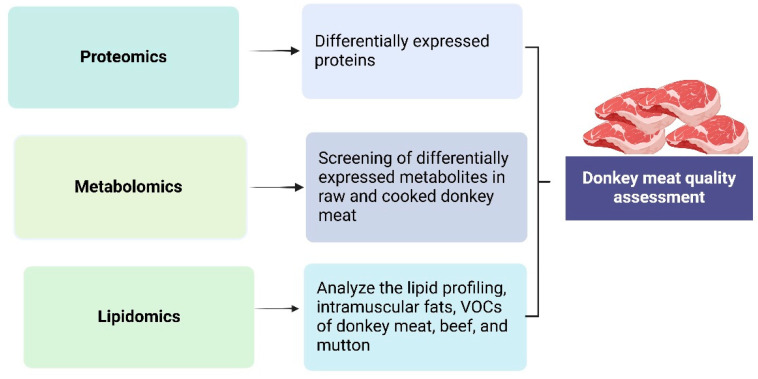
The application of various omics technologies for donkey meat quality assessment. The figure illustrates proteomics analysis [93,94,95], lipidomic applications [78,96,99], and metabolomics [100] of donkey meat.

Meat tenderness, a critical quality parameter for consumer acceptance [107], is primarily influenced by muscle proteolytic potential and myofibrillar protein degradation [94,95]. Electrical stimulation has been shown to accelerate acid release, modify muscle fiber structure, and calpain degradation, thereby enhancing meat tenderness [96]. Tenderness typically correlates positively with pH values [108]. Donkey meat is weakly alkaline after slaughter. Tissue enzymes decompose glycogen and phosphorus-containing compounds to produce lactic acid and phosphoric acid, making the meat acidic, enhancing connective tissue softening, and improving meat tenderness [109]. LC-MS-based metabolomics has identified key metabolites including inosine, adenine, N-acetylhistidine, and citric acid, whose levels correlate with pH and shear stress, with optimal tenderness observed at 4 h postmortem [110] (Figure 1).

### 4.4. Genomic and Transcriptomic Approaches for Screening Potential Candidate Genes Associated with Meat Phenotypic Traits

The molecular underpinnings of donkey meat quality represent a complex interplay between genetic regulation and biochemical processes. The inverse relationship between muscle fiber proliferation and meat quality has been well established, as increased fiber density correlates with elevated drip loss and compromised tenderness. Recent genomic investigations have elucidated several candidate genes associated with these quality-determining phenotypic traits [111,112]. Consistently, Sun et al. [93] identified a comprehensive network of genes implicated in myofibrillar structure and development, encompassing five troponin variants (*TNNC1*, *TNNC2*, *TNNI1*, *TNNI2*, and *TNNT1*), seven myosin genes (*MYBPC1*, *MYBPC2*, *MYH2*, *MYH7*, *MYL1*, *MYL2*, and *MYL3*), and two tropomyosin genes (*TPM1* and *TPM3*). These genes serve a dual function—encoding critical myofibrillar proteins while concurrently orchestrating skeletal muscle development in donkeys. Subsequent dual luciferase assays via psiCheck2 vector transfection confirmed *TPM3*’s role in eca-miR-1-targeted muscle development, thus substantiating eca-miR-1’s significance in myogenesis [113].

Furthermore, Chai et al. [113] demonstrated pronounced differential gene expression profiles across various muscle types in Dezhou donkeys. Notably, *ENO3*, *MYH1*, *MYH4*, *TNNI3*, *PGK1*, and *ALDOA* exhibited significant variation, with these genes fundamentally linked to muscle fiber composition, meat tenderness, and glucose metabolic pathways [114]. Transcriptome analysis by Yu et al. [115] identified candidate long non-coding RNAs (lncRNAs) that regulate key skeletal muscle development genes including *DCN*, *ITM2A*, *MUSTN1*, and *ARRDC2* [93]. Similar genomic screening studies have identified polymorphisms and related genes associated with body characteristics of Yangyuan donkey [116] and Xinjiang donkey [117]. Although lncRNAs have been widely studied in livestock species, the regulation mechanism of testicular development in donkeys is still lagging behind. Previous transcriptome studies on the testicular tissue of Dezhou donkeys have identified differentially expressed lncRNAs, revealing the molecular mechanism of mRNA and lncRNA synergistically regulating the post-transcriptional expression of spermatogenesis-related genes [118].

Furthermore, IMF constitutes a critical determinant of meat organoleptic properties, as its inherent softness relative to muscle fibers directly enhances tenderness and juiciness [119]. Consequently, IMF serves as an essential index for quality assessment. Peng Y’s [120] research demonstrated lncRNAs’ crucial regulatory function in adipogenesis, with *SCD* and *THRSP* emerging as potential master regulators in this process. Li et al. [121] further elucidated fat deposition mechanisms by identifying 167 DEGs involved in lipid metabolism. Key genes have been identified as potential candidates for regulating intramuscular fat, though specific mechanisms warrant further investigation. Additionally, Li et al. identified differentially expressed genes including *LEPR*, *CIDEA*, *DLK1*, and *DGAT2* related to adipocyte differentiation in the longissimus dorsi of Guangling donkey, alongside candidates such as *EEF2*, *DDX49*, and *GAP43* critical for IMF regulation. Interestingly, volatile compounds have been implicated in IMF deposition through their participation in adipogenesis signaling pathways [119]. Complementary studies [122] revealed DEGs associated with carbohydrate enrichment, lipid metabolism, endocrine signal transduction, and cellular processes that potentially enhance meat tenderness. Furthermore, circular RNA (circRNA) expression analysis in longissimus dorsi muscle demonstrated their function as miRNA sponges in lipid metabolism regulation, thereby influencing IMF deposition and, ultimately, meat quality [123].

Our research consortium has conducted comprehensive genomic analyses to elucidate the genetic architecture underlying economically significant phenotypic traits in donkeys. Through genome-wide association studies (GWAS), candidate gene approaches, and transcriptomic methodologies, we have identified numerous loci significantly associated with production traits pertinent to meat quality and quantity, including vertebral count variability [124,125], carcass yield parameters [126,127], and metrics related to growth rate and morphological conformation [117,128]. The identified genetic variants exert multifaceted effects on meat production traits through diverse physiological mechanisms. Lipid metabolism genes, including *SCD*, *LEPR*, and *CIDEA*, demonstrate significant associations with intramuscular adipose tissue deposition and adipogenic processes, which contributes substantially to organoleptic properties, enhancing both flavor complexity and textural characteristics of the meat. We have further characterized genes implicated in skeletal development and somatic growth, including *NCAPG*, *Wnt7a*, *BMP7*, *DCAF7*, *HOXC8*, *PRKG2*, and *LCORL*, which exhibit significant associations with body dimensional traits and are consequently determinants of carcass yield. Additionally, thoracic conformation genes, specifically *NFATC2* and PROP1, demonstrate associations with chest circumference and cardiac girth measurements that contribute to the overall skeletal framework supporting optimal musculature development, providing the foundational architecture for superior muscular development and consequent meat quality attributes. Further details on the potential genes linked to these phenotypic traits of meat production in donkeys have been provided in Table 2.

## 5. Authentication Methods for Donkey Meat

The adulteration of premium meat products with lower-quality alternatives has necessitated the development of robust authentication methods [143,144,145,146]. Modern analytical technologies encompass multiple sophisticated approaches for detecting meat fraud. Species-specific PCR assays utilize unique DNA sequences to amplify and detect fragments indicative of adulterated meat sources [147,148,149]. Electronic nose technology identifies meat adulteration by detecting and analyzing the electrical signals generated by volatile compounds, creating characteristic odor profiles for comparison [150,151]. Liquid chromatography-tandem mass spectrometry (LC-MS/MS) combined with multispectral imaging offers a dual approach: protein component identification coupled with structural distribution visualization to precisely detect meat adulteration [152,153]. Additionally, ¹H-NMR metabolomics enables the identification of key biomarkers such as lactic acid, creatine, and choline, effectively differentiating between white and red meat varieties, including chicken, donkey, and beef [154].

Recent innovations include recombinase polymerase amplification (RPA) combined with CRISPR/Cas12a for meat species identification. Following parameter optimization, this isothermal approach specifically detects various meat species with a sensitivity of 1 × 10^0^ /μL within 60 min. In this system, CRISPR/Cas12a employs guide RNA to recognize target meat DNA sequences, activating Cas12a nuclease activity. When coupled with lateral flow dipstick (LFD), this technology enables visual result interpretation, offering a rapid, portable, and user-friendly meat authentication method [155]. Furthermore, real-time exponential recombinase amplification (ERA) and lateral flow strip ERA assays targeting mitochondrial genes (ATPase 6 or ND2) have been developed for horse, donkey, and porcine component detection. These assays demonstrate optimal performance at 39 °C and 37 °C, respectively, yielding results within 25 min. The methods exhibit high specificity with detection limits of 10 pg genomic DNA per reaction and 0.1% target meat, showing concordance with national standard PCR methods while significantly reducing analysis time [144].

While PCR-based technologies remain prevalent for donkey meat authentication, they present several implementation challenges. Sample quality significantly impacts test outcomes, as contamination, degradation, or insufficient DNA extraction can lead to false negative or false positive results. For phylogenetically related species, primer design presents technical difficulties and increased costs. Moreover, extremely low adulteration ratios may escape detection due to insufficient target DNA concentration.

## 6. Influencing Factors of Donkey Meat Quality and Nutritional Value

### 6.1. Breed Variation

Comparative studies between North African donkey (NAD) and Masri donkey (MD) breeds have revealed distinct quality characteristics. NAD meat exhibits superior final weight, IMF content, and hue value, while MD meat demonstrates higher cold dressing percentage and cooking loss value, suggesting differential tenderness properties [156]. Analysis of coastal Dinara and Easter donkeys showed significant variations in carcass weight and slaughter rates, with boneless meat proportions of 26.18% and 28.27%, respectively. While breed differences significantly affected meat piece quality, they showed minimal impact on color, pH, and composition, but notably influenced milk characteristics and n-6/n-3 PUFA ratios [60]. Intra-breed variations are also significant, as demonstrated in Dezhou donkey lines (SanFen and WuTou), where 38 volatile compounds showed distinct profiles. SanFen meat exhibited higher ketone and alcohol content but lower aldehyde levels compared to WuTou meat [104].

### 6.2. Age Effects

Age significantly influenced meat quality parameters in equine species. A study investigating 16 Martina Franca foals revealed that 12-month-old specimens exhibited higher carcass weights and elevated muscle glycogen content, whereas meat from 8-month-old foals demonstrated superior tenderness [52]. Both age groups yielded meat rich in essential amino acids and unsaturated fatty acids [90]. Further research on ‘Galician Mountain’ foals identified significant age-dependent variations: carcass weight increased with maturation; 8-month-old meat presented higher cholesterol concentrations and greater colorimetric brightness values. Regarding fatty acid profiles, 8-month-old specimens contained significantly higher n-3 polyunsaturated fatty acid concentrations and lower n-6 fatty acid content, indicating substantial differences in both organoleptic qualities and nutritional composition between age groups [156,157].

### 6.3. Feeding Management Impact

Different feeding regimens significantly influence meat quality parameters [158]. Intensive feeding systems yield higher IMF and SFA content with enhanced tenderness, while extensive feeding promotes elevated protein and unsaturated fatty acid levels, including n-3 essential fatty acids. GMF donkeys under free extensive breeding show higher PUFA content, while semi-extensive systems favor MUFA accumulation [58,159].

Dietary supplementation with grass, flaxseed, and polyunsaturated fatty acid-rich oils enhances muscle PUFA and conjugated linoleic acid content while reducing saturated fatty acid proportions. Notably, grass-derived vitamin E contributes to extended shelf life [160,161]. Yeast polysaccharide supplementation positively impacts feed intake and meat quality [9]. Artificial lactation, compared to natural methods, demonstrates superior outcomes in foal growth rates and meat quality parameters, including protein and IMF content [162,163].

### 6.4. Storage and Processing Considerations

Postmortem aging and storage significantly affect meat quality through oxidation and nitrification involving ROS and RNS, affecting fat and myofibrillar protein [164,165]. The results showed that the tenderness, PUFA content, and VOC composition of donkey meat changed significantly within 15 days after slaughter [106]. After 8 days and 15 days of aging, the content of PUFA increased significantly, and some PUFA played a key role as a precursor of antithrombotic factors [166]. The furan produced by amino acids increased significantly during aging, which may lead to Maillard reaction due to the increase of free amino acids during storage, resulting in the change of VOC composition [167,168]. Quality preservation strategies include low-voltage electrical stimulation for rapid acid removal [169]. The processing of dry-cured donkey leg meat showed that pH was stable, water activity decreased, chloride content increased, free amino acid and fatty acid composition increased, and was accompanied by the development of complex volatile compounds [79].

## 7. Research Gaps

The current state of donkey meat research exhibits several critical knowledge gaps across four key domains: genetics, technology, industry standards, and breeding programs. This review identifies and analyzes these limitations to guide future research efforts. In the genetics domain, the functional mechanisms of candidate genes affecting meat quality remain poorly characterized, particularly regarding complex interactions and regulatory networks. Limited studies exist examining the association between genetic markers and meat quality traits across different donkey breeds and populations, impeding progress in directional breeding. The relationship between muscle fiber type composition and resultant meat quality characteristics is insufficiently documented. Environmental factors and stress responses affecting meat quality at the molecular level require further investigation. Regarding technology, comprehensive research exploring the effects of diverse feeding strategies on meat quality at the molecular level is lacking. Multi-omics approaches to investigate post-slaughter biochemical changes and their effects on meat quality have yielded limited results, restricting our understanding of the meat maturation process. In the industry sector, the absence of standardized identification methods and quality evaluation criteria for donkey meat processing complicates quality control and fraud prevention efforts. For breeding programs, despite the identification of numerous genetic markers associated with meat quality, their practical application is hindered by inadequate targeted breeding programs. A deficiency of breed-specific breeding programs limits the potential for genetic improvement across different donkey populations. Addressing these research gaps would significantly advance donkey meat production and quality improvement, ultimately benefiting both producers and consumers in this emerging market sector.

## 8. Conclusions

This review highlights the significant achievements in the analysis of the molecular basis of donkey meat characteristics by using various omics techniques. The combined use of proteomics, lipidomics, and metabolomics provides key clues for understanding the unique nutritional components and quality characteristics of donkey meat. Genomic and transcriptomic analyses have accurately mapped key genes closely related to growth traits and meat quality, such as *ACTN3*, *TPM2*, *TPM3*, *NCAPG*, and *LCORL*, which are critical for muscle development, IMF deposition, and tenderness. Lipidomics studies identified lipid-based biomarkers, including TG (16:1_18:1_18:2) and PC (18:3e_16:0), which correlate with flavor retention and oxidative stability. Metabolomic profiling revealed metabolites such as L-glutamic acid, γ-aminobutyric acid, and maltotriose as potential biomarkers for flavor differentiation between raw and cooked donkey meat. Additionally, genes like *SCD*, *BMP7*, *NR6A1*, *Wnt7a*, *HOXC8*, *LCORL*, *LEPR*, and *CIDEA* have been implicated in adipogenesis and IMF regulation, offering actionable targets for genetic improvement programs. These findings underscore the potential of molecular markers, such as the aforementioned genes, lipids, and metabolites, to serve as robust biomarkers for meat quality assessment, authentication, and breeding strategies. Future research should prioritize validating these biomarkers across diverse donkey populations and integrating them into standardized quality evaluation frameworks to enhance the precision and sustainability of the donkey meat industry. By bridging these gaps, the industry can achieve significant advancements in production efficiency, product authenticity, and consumer satisfaction.

## Figures and Tables

**Table 1:** Comparative analysis of nutritional characteristics and mineral content of different meat.

Nutrients	Donkey Meat [58,59,60,61,62]	Beef [63,64,65,66]	Pork [67,68,69,70,71,72]	Sheep [58,73,74,75,76,77]
Protein (g/100g)	23.56	23.50	18.60	20.70
Fat (g/100g)	1.77	4.53	23.80	8.85
Ash%	1.13	1.13	0.90–1.00	1.62
Vitamin B12 (μg/100 g)	1.90	6.53	1.00	2.08
Sodium (mg/100g)	36.80–83.60	64.80	53.00	85.70
Phosphorus (mg/100g)	185.00–335.00	182.00	190.00	611.36
Iron (mg/100g)	2.86–4.77	1.76	1.05	2.93
Zinc (mg/100g)	2.99–4.71	3.27	1.90	3.23
Calcium (mg/100g)	7.95	3.72	7.00	21.34
Potassium (mg/100g)	353.00	391.00	330.00	280.00
Cholesterol (mg/100 g)	66.70	63.00	77.00	133.28
Polyunsaturated fatty acids (PUFA)/saturated fatty acids	0.73	0.15	0.29	0.09

**Table 2:** Potential genes and their association with meat quality phenotypic traits of donkeys.

Genes	Association with Meat Quality and Growth Traits	Breed	Omics Techniques /Instruments	Reference
*ACTN3*, *TPM2*, *TPM3*	✧ Involved in fibrogenesis, influence muscle tenderness, play roles in growth, development, and muscle characteristics	Dezhou Donkeys	Transcriptomics	[8]
*NCAPG*, *LCORL*	✧ Related to growth, development, and body size traits	Guanzhong, Taihang, Dezhou, Huaibeihui, Biyang, and Qingyang Qinghai, Guoluo, Xinjiang, XizangGuanzhong, Taihang, Dezhou, Huaibeihui, Biyang, Qingyang	Genomics	[9]
*KRT10*, *KRT1*, *CLDN9*	✧ Associated with skin thickness and muscle development	Dezhou Donkeys	Transcriptomics	[11]
*ARF6*, *IQGAP*, *AGPAT1*	✧ Related to meat quality traits (meat tenderness)	Dezhou Donkeys	Proteomics	[93]
*MYH1*, *MYH7*, *TNNC1*	✧ Involved in skeletal muscle growth and development	Dezhou Donkeys	Transcriptomics	[113]
*NFATC2*, *PROP1*	✧ Linked to chest circumference and heart girth	Xinjiang donkeys	Genomics	[117]
*SCD*, *LEPR*, *CIDEA*	✧ Responsible for intramuscular fat deposition, adipogenesis, and muscle tenderness	Guangling donkeys	Transcriptomics	[121]
*miR-429*, *miR-224*, *miR-125a-5p*, *miR-223*	✧ Facilitate improvement of intramuscular fat content	Liaoxi donkey	Transcriptomics	[123]
*DCAF7*	✧ Related to the number of thoracolumbar vertebrae, carcass traits, and hide weight	Dezhou donkey	Targeted sequencing Sanger sequencing	[124]
*PRKG2*	✧ Carcass weight, number of thoracic vertebrae ✧ The number and the length of lumbar vertebrae ✧ The total number of thoracolumbar vertebrae	Dezhou donkey	Targeted sequencing	[125]
*NLGN1*, *DCC*, *SLC26A7*, *LCORL*, *BMP7*, *Wnt7a*	✧ Involved in regulating Wnt and TGF − β signaling pathways related to embryonic development or bone formation; associated with the number of thoracic and lumbar vertebrae	Dezhou donkeys	Genomics	[126]
*NR6A1*	✧ Associated with lumbar vertebrae number, the total number of thoracolumbar, body size and carcass weight	Dezhou donkeys	Genomics	[127]
*SMPD4*, *RPS6KA6*	✧ Related to body size and growth traits	Yangyuan donkeys	Genomics	[128]
*NKX1-2*	✧ Correlated with body length, thoracic girth, hide weight, body height and carcass weight	Dezhou donkeys	Genomics	[129]
*LTBP2*	✧ Associated with thoracic vertebrae number, lumbar vertebrae number, chest circumference, and carcass traits	Dezhou donkeys	Genomics	[130]
*HOXC8*	✧ Related to carcass weight and lumbar vertebrae length	Dezhou donkeys	Genomics	[131]
*LCORL*	✧ Associated with body and hide weight; related to higher body height, body length, chest circumference, and hide weight	Dezhou donkeys	Targeted sequencing	[132]
*IGF-1*, *IGF-2*	✧ Linked to chest circumference, chest depth, rump height, and body length	Dezhou donkeys	TranscriptomicsGenomics	[133,134]
*TBX3*	✧ Involved in growth and biometric measurement traits (body weight, length, height, chest depth and circumference, hucklebone width, rump length)	Dezhou donkeys	Genomics	[135]
*CDKL5*	✧ Associated with body size traits (chest circumference and depth, rump width, body length)	Dezhou donkeys	Genomics	[136]
*ACSL1*	✧ Related to body size traits (withers height, body length, rump width, body weight)	Dezhou donkeys	Transcriptomics, Genomics	[137]
*ACSL3*	✧ Linked to growth traits (body weight, chest width, chest depth)	Dezhou donkeys	Genomics	[138]
*ACTN1*, *CDON*, *FMOD*, *BMPR1B*	✧ Involved in growth and skeletal muscle development	Dezhou donkeys	Transcriptomics	[139]
*LCORL/NCAPG*, *FAM184B*, *TBX3*, *IHH*	✧ Related to body size traits (body height)	Biyang, Dezhou, Guangling, Hetian, Jiami, Kulun, Qingyang, Turfan, Tibetan, Xinjiang, Yunnan, Zamorano~Leonés and Andalusian	Whole genome resequencing technology	[140]
*NCAPG*, *LCORL*, *CYP4A11*	✧ Linked to body height	Liangzhou donkeys	Whole genome resequencing technology	[141]
*SPAG8*, *RPL27A*, *TPM1*,	✧ Involved in intramuscular fat deposition, adipogenesis, muscle tenderness, and bone development	Liaoxi donkeys	Proteomics	[142]

## Data Availability

Not applicable.
